# Iron Overload in Diabetic Retinopathy: A Cause or a Consequence of Impaired Mechanisms?

**DOI:** 10.1155/2010/714108

**Published:** 2010-08-08

**Authors:** Andreea Ciudin, Cristina Hernández, Rafael Simó

**Affiliations:** CIBER de Diabetes y Enfermedades Metabólicas Asociadas (CIBERDEM), Instituto de Salud Carlos III (ISCIII), Unidad de Diabetes y Metabolismo, Instituto de Investigación Hospital Universitario Vall d'Hebron, Paseo Vall d'Hebron 119-129, 08035 Barcelona, Spain

## Abstract

Iron is an essential ion for life, playing a central role in many metabolic processes. The most important property of free iron is its capacity to be reversibly oxidized and reduced, but at same time this make it highly pro-oxidant molecule. In this regard, iron is able to generate powerful reactive oxygen species (ROS). For this reason, careful control on iron availability is central to the maintenance of normal cell function in the retina. In the diabetic eye there is an impairment of iron homeostasis, thus leading to iron overload. The mechanisms involved in this process include: (1) Destruction of heme molecules induced by hyperglycemia (2) Intraretinal and vitreal hemorrhages (3) Overexpression of the renin-angiotensin system. The main consequences of iron overload are the following: (1) Retinal neurodegeneration due to the increase of oxidative stress (2) Increase of AGE-RAGE binding (3) Defective phagocytosis of retinal pigment epithelium, which generates the accumulation of autoantigens and the synthesis of proinflammatory cytokines. Further studies addressed to explore not only the role of iron in the pathogenesis of diabetic retinopathy, but also to design novel therapeutic strategies based on the regulation of iron homeostasis are needed.

## 1. Introduction

Diabetic retinopathy (DR) is the leading cause of blindness in working-age individuals in developed countries [1]. DR classically has been considered as a microcirculatory disease of the retina due to the deletereous metabolic effects of hyperglycemia *per se* and the metabolic pathways triggered by hyperglycemia on retinal capillaries [2]. In recent years, evidence has emerged showing that retinal neurodegeneration is an early event in DR and is already present before any microcirculatory abnormalities can be detected in ophthalmoscopic examination [3–7]. However, this subject is still controversial, since not all of the studies evidence retinal neurodegeneration in the diabetic retina [8]. Alterations contributing to oxidative stress and downregulation of antioxidative enzymes play an important role in the pathogenesis of DR [9, 10]. Oxidative stress is considered to be one of the crucial contributors to the pathogenesis of DR and it is highly interrelated with other biochemical imbalances (i.e., increase in the polyol, PKC, hexosamine, and advanced glycation end-products [AGEs] pathways), that lead to structural and functional changes such as accelerated loss of capillary cells in the retinal microvasculature, increased vascular permeability, and increased VEGF formation [9–13].

Iron is an essential ion for life, playing a central role in many metabolic processes. Many enzymes on important metabolic pathways are iron dependent, thus making iron necessary for essential processes such as DNA synthesis, myelin production, and synthesis of the ATP (adenosine triphosphate), as well as several neurotransmitters (i.e., serotonin, dopamine) [14–16]. The most important property of free iron is its capacity to be reversibly oxidized and reduced, but at the same time this makes it a highly pro-oxidant molecule. In this regard, iron is able to generate powerful reactive oxygen species (ROS) [17, 18]. Therefore, the maintenance of iron homeostasis in the organism is crucial, and high levels of free iron could be harmful. 

 In the human retina, iron levels increase with age in both men and women. However, women have significantly more retinal iron than men at all ages, in spite of having a higher incidence of anemia, which suggests tissue-specific mechanisms of iron regulation [19]. Abnormalities in local iron homeostasis have been implicated in several degenerative diseases, including Parkinson's, Alzheimer's, and age-related macular degeneration, where it has been hypothesized that oxidative stress contributes to cell death [20–22]. In addition, iron participates in other ocular diseases such as glaucoma and cataract [23, 24]. However, the potential role of dysregulation of iron metabolism in the pathogenesis of DR remains to be elucidated. Here we present an overview of the intricate network of proteins involved in retinal iron handling, and we discuss evidence which suggests that iron may contribute to retinal degeneration observed in DR.

## 2. Iron Homeostasis in the Retina

Since iron is highly toxic due to its ability to generate free radicals, homeostatic mechanisms maintain iron levels by regulation of the proteins involved in iron import (transferrin, transferrin receptor, divalent metal transporter-1), storage (ferritin), and export (ceruloplasmin, hephaestin, ferroportin, and hepcidin) [16, 25–34]. The opposing requirements and toxicities of iron are managed by an iron-responsive mechanism of posttranscriptional regulation of key iron-handling proteins [35]. This regulation allows individual cells to regulate iron uptake, sequestration, and export according to their iron status. Iron-regulatory proteins (IRPs) register intracellular iron status and, in cases of intracellular iron deficiency, bind to iron-responsive elements (IREs) on the mRNA of the regulated protein [36–39].

Iron circulates in the blood stream by being incorporated in the heme molecule of hemoglobin and mioglobin, and most nonheme iron is bound to transferrin, a protein capable of binding two molecules of ferric iron. Iron uptake by cells involves the transferrin binding to its receptor (Tf-R) and subsequent endocytosis. After acidifcation of the endosome, transferrin releases its iron and is recycled to the membrane where it is released to the extracellular space. Iron in the endosome is then transported out through ferroportin or by divalent metal transporter-1 (DMT1).

Transferrin is present in the vitreous fluid of rabbits at a higher relative concentration found in the plasma or in the aqueous humor [40]. In fact, transferrin makes up about 25% of the total protein in the rabbit's vitreous humor [41]. In animal models and the human retina, the main site of transferrin synthesis is the retinal pigment epithelium (RPE) [33, 40, 42, 43]. 

Transferrin may protect the retina from the potentially toxic effects of unbound iron, because iron bound to transferrin does not cause oxidative stress [44]. Transferrin probably helps to transport iron to photoreceptors through their Tf-R [33]. Finally, transferrin may also have neurotrophic effects that are essential for normal retinal functioning [45]. In the rat, retina Tf-R has been detected in the RPE, the inner segments of photoreceptors, the outer plexiform layer, inner nuclear layer, inner plexiform layer, and in the ganglion cell layer [33]. Tf-Rs are located on both the basolateral and apical surfaces of RPE cells, suggesting that there is a bidirectional iron stream in the blood-retinal barrier depending on the iron status in the epithelial cells [16, 33].

Once into the cell, the iron is rapidly uptaken by cytosolic ferritin, a protein capable of incorporating 4.500 iron molecules. Ferritin is composed of 24 subunits of two chains: H-ferritin (heavy chain, or “heart ferritin”) which possesses a ferroxidase function which reduces the ferric form to the ferrous one, mainly localized in the heart, and L-ferritin (“light” or “liver” ferritin) which does not have ferroxidase activity [31]. The ability of cells to store and retrieve iron from ferritin is dependent on the ratio of H : L ferritin chains, but the mechanisms that regulate this ratio are not fully understood. H-ferritin is not only responsible for iron oxidation and uptake, but also has other functions such as reducing the cell proliferation rate and apoptosis [46, 47]. Another form of ferritin, mitochondrial ferritin, has been identified. Mitochondrial ferritin is 80% homologous to H-ferritin found in the cytoplasm and stores iron more efficiently than cytoplasmic ferritin [48, 49]. In the murine retina, mitochondrial ferritin has been found in the photoreceptor inner segments and diffusely throughout the inner retina [50]. 

 By reducing the intracellular level of free iron, ferritin is capable of reducing oxidative stress. There are some factors such as ascorbic acid (Vitamin C), alpha-lipoic acid, or UVB irradiation that can affect iron metabolism [51]. Ascorbic acid is present in the retina at a high concentration compared with its presence in other human organs, and it is able to protect the retina against oxidative damage [52–54]. In this regard, we have recently found *∼*20-fold higher levels of ascorbic acid in the vitreous fluid than in serum. In addition, the vitreous fluid from PDR patients contained a significant lower amount of ascorbic acid in comparison with vitreous samples from nondiabetic subjects [55]. Moreover, it has been demonstrated that ascorbic acid causes large increases in ferritin synthesis and increased loading of iron into ferritin in cultured epithelial cells of the lens [56, 57]. Therefore, the effect of ascorbic acid in iron metabolism contributes to its antioxidant properties, and the reduced levels detected in the eyes of diabetic patients could be involved in the pathogenesis of DR. 

 The iron which is not used by the cell needs to be returned to the blood stream. Only ferrous iron can pass through the plasma membrane into the blood, and only ferric iron can be incorporated into transferrin [58]. Therefore, the iron is transported out of the cell bound to ferroportin (a cell membrane protein), and it is then oxidized by the ferroxidases ceruloplasmin and hephaestin, thus making it available to be bound to transferrin.

## 3. Iron-Dependent Regulation of Retinal Functions

The RPE constitutes the outer blood-retinal barrier and regulates the flow of iron between the choroidal vasculature and the outer retina. Of all the retinal cell types, RPE cells are theoretically the most susceptible to oxidative damage because of their proximity to the choriocapillaries. In fact, in the human retina, the highest levels of iron are found in the choroid, RPE, and photoreceptor segments [50]. However, other cell types such as pericytes, endothelial cells, retinal Muller cells, ganglion cells, and astrocytes can be, affected even earlier than the photoreceptors and RPE cells by oxidative damage [12]. Iron in the eye is important for the phototransduction cascade. Indeed, iron is an essential cofactor for the enzyme guanylate cyclase, which synthesizes cGMP, the second messenger in the phototransduction cascade [59]. In addition, isomerization of the all transretinal within the retinal pigment epithelium (RPE) in the visual cycle requires iron for the activation of RPE65, an enzyme involved in the visual cycle pathway [60]. 

 The citosolic aconitase system, a dual-function protein involved in the metabolic regulation of iron that is found in all mammalian cell types studied, is located in the RPE of the retina [51]. When iron is scarce, c-aconitase functions as an iron regulatory protein (IRP) controlling the translation of numerous proteins. However, when iron is abundant, the IRP triggers aconitase activity and regulates L-glutamate production, a neurotransmitter involved in retinal neurodegeneration [61]. Thus, cultured lens epithelial cells (LECs), retinal pigment epithelial (RPE) cells, and retinal neurons synthesize and secrete L-glutamate, and this process is regulated by iron by way of its effect on c-aconitase [61]. Elevated levels of glutamate in the retina have been found in experimental models of diabetes, as well as in the vitreous fluid of diabetic patients with PDR [62–65]. The heme oxygenase (HO) system acts as an antioxidant. There are 2 main isoforms of HO: HO-1, a “heat shock protein”, which is very sensitive to oxidative stress [66], and HO-2, which is expressed constitutionally in the endothelial, neural, retinal and testicular cells [67]. HO-1 is an inducible enzyme whose activity increases in response to iron as well as heme, light, oxidative stress, and inflammation. It degrades heme to iron, carbon monoxide (CO), and biliverdin. The release of iron upregulates the synthesis of ferritin as a cytoprotective mechanism (see below). CO has important roles in vasodilatation, and biliverdin is subsequently converted to the antioxidant bilirrubin. It has been demonstrated that increasing HO-1 promoter activity in RPE cells could trigger a protective response [68]. In the retina, overexpression of HO-1 in photoreceptor cells provided protection from light damage [69]. In murine models, HO-1 and HO-2 were localized in the outer segment of the photoreceptor layer, inner plexiform layer, ganglion cell layer, glial fibres, and capillary endothelium [70]. 

 However, during hemorrhage, the excesive generation of iron and bilirrubin, that is neurotoxic, has deleterious consequences. 

 Severe hypoxia due to capillary occlusion is the main condition for the initiation of neovascularization in PDR. Hypoxia upregulates the expression of angiogenic factors directly or through the hypoxia-inducible factor (HIF-1). HIF-1 activates several genes related to iron metabolism such as HO-1, transferrin, transferrin receptor, and ceruloplasmin [71–73]. In addition, it has been recently demonstrated that ischemic preconditioning of the retina is highly effective in preventing subsequent injury caused by iron-dependent free radical burst after prolonged ischemia. This protection appears to be provided by increased ferritin levels [74].

## 4. Disruption of Iron Homeostasis and Oxidative Damage in DR

It has been demonstrated that intravitreal levels of iron in PDR are 2.5 times the normal levels [75]. In addition, trasferrin concentrations have been found elevated in the vitreous fluid and retinal membranes from patients with proliferative vitreoretinopathy diseases including PDR [76, 77].

**Figure 1:**
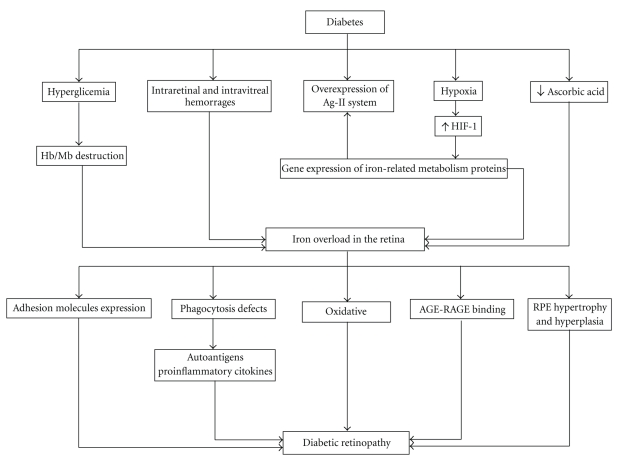
Scheme illustrating how diabetes influences iron metabolism in the retina and the pathogenic consequences.

There are several mechanisms that could explain iron overload in diabetic eyes (Figure 1). First, it has been demonstrated *in vitro* that hyperglycaemia causes a complete destruction of heme molecules from hemoglobin and myoglobin, releasing free iron into the interstitial space [78, 79]. Second, intraretinal and vitreal hemorrages could contribute to iron overload in PDR. Finally, angiotensin II stimulates the local gene expression of proteins related to iron metabolism (TfR, DMT1, ferroportin, and hepcidin) in the rat kidney, thus contributing to the production of high levels of iron transporters and facilitating iron uptake by the cells [80, 81]. In this regard, it is worth mentioning that the major components of the renin-angiotensin system, including angiotensin II, have been identified in glial cells, neurons, and blood vessels from murine retinas [82–84] and are overexpressed in diabetic rats [85, 86]. In human beings, vitreal concentrations of prorenin, renin, and Ang II are elevated in patients with DR [87, 88]. In addition, proteomic analysis of vitreal samples taken from patients with diabetes revealed that angiotensinogen was found in greater concentration in samples taken from those with PDR, compared with those with no DR or non-diabetic control subjects [89]. 

The consequences of iron overload in the diabetic eye are complicated to evaluate because, as mentioned above, there are multiple forms of iron with different reactivity and several proteins that modulate their levels and actions. However, among the potential mechanisms of iron-induced damage, it seems that oxidative damage is the most important (Figure 1). 

Increased intraocular levels of iron cause oxidative damage to photoreceptors with greater damage to cones than rods [90]. In addition, it has been shown that iron chelation protects the RPE cells against cell death induced by oxidative stress [91, 92]. In the retina of human donors with DR high levels of peroxidized lipids in Bruch's membrane promoted by the local ferric iron involved have been demonstrated [93]. This happens also in the eyes of patients suffering from vitreal bleeding in the course of PDR, after which there is an important reactivation of superoxide generation catalyzed by the locally released free iron [94]. In addition, it should be noted that iron ion catalyses the binding of the AGEs (advanced glycation end products) to the specific receptor, that is, a crucial step in the pathogenesis of the DR [95]. 

It has been demonstrated that impaired retinal iron homeostasis is associated with defective phagocytosis in both murine models [96] and in ARPE-19 cell cultures [97]. An impairment of phagocytosis has been described in long-term diabetes [98] and, therefore, it is possible that this could also happen to RPE cells. Thus, iron overload could contribute to the phagocytosis defect associated with diabetes. This defect implies a delayed and impaired phagocytosis of both the apoptotic cells and the local detritus, which generates the accumulation of autoantigens and the synthesis of proinflammatory cytokines.

As mentioned above, HO may respond to oxidative stress and upregulation of the HO system (HO-1 and HO-2) in rats with streptozotocin- induced diabetes has been demonstrated [70]. In diabetic rats, increased retinal HO-1 mRNA expression has been shown to be preventable with antioxidant therapy [99], and HO-1 overexpressing neurons have shown reduced levels of apoptosis [100]. However, in human eyes with long-term diabetes, reduced HO-1 mRNA expression in RPE cells has been demonstrated [101], thus suggesting that increased HO activity induced by diabetes is dependent on diabetes duration. 

Although the HO system has been generally accepted as having an antioxidant role in several tissues, HO also could exhibit pro-oxidant activity in the vascular endothelial cells. For example, it has been demonstrated that in the endothelial cells, HO increases the expression of nitric oxide (NO), endothelin-1, and VEGF [102–104], all of which are relevant factors in the pathogenesis of PDR [2].

In cellular cultures, free iron stimulates the expression of adhesion molecules and monocyte endothelial adhesion, [105–107] key steps in the development of DR. Finally, in murine models, iron overload is associated with RPE hypertrophy and hyperplasia due to the stimulation of citosolic-aconitase system, which acts as an enzyme initiating the proliferation cascade [108].

In summary, careful control of iron availability is central to the maintenance of normal cell functions. Iron overload seems to be caused by several processes involved in the pathogenesis of DR. However, at the same time iron causes retinal damage mainly by increasing oxidative stress. Further studies addressed to exploring the role of iron in the pathogenesis of DR are necessary not only to improve our knowledge on this issue, but also to design novel therapeutic strategies based on the regulation of iron proteins.

## References

[1] Congdon NG, Friedman DS, Lietman T (2003). Important causes of visual impairment in the world today. *Journal of the American Medical Association*.

[2] Simó R, Carrasco E, García-Ramírez M, Hernández C (2006). Angiogenic and antiangiogenic factors in proliferative diabetic retinopathy. *Current Diabetes Reviews*.

[3] Carrasco E, Hernández C, Miralles A, Huguet P, Farrés J, Simó R (2007). Lower somatostatin expression is an early event in diabetic retinopathy and is associated with retinal neurodegeneration. *Diabetes Care*.

[4] Carrasco E, Hernández C, de Torres I, Farrés J, Simó R (2008). Lowered cortistatin expression is an early event in the human diabetic retina and is associated with apoptosis and glial activation. *Molecular Vision*.

[5] Garcia-Ramírez M, Hernández C, Villarroel M (2009). Interphotoreceptor retinoid-binding protein (IRBP) is downregulated at early stages of diabetic retinopathy. *Diabetologia*.

[6] Barber AJ, Lieth E, Khin SA, Antonetti DA, Buchanan AG, Gardner TW (1998). Neural apoptosis in the retina during experimental and human diabetes: early onset and effect of insulin. *Journal of Clinical Investigation*.

[7] Antonetti DA, Barber AJ, Bronson SK (2006). Diabetic retinopathy: seeing beyond glucose-induced microvascular disease. *Diabetes*.

[8] Kern TS, Barber AJ (2008). Retinal ganglion cells in diabetes. *Journal of Physiology*.

[9] Madsen-Bouterse SA, Kowluru RA (2008). Oxidative stress and diabetic retinopathy: pathophysiological mechanisms and treatment perspectives. *Reviews in Endocrine and Metabolic Disorders*.

[10] Jarrett SG, Lin H, Godley BF, Boulton ME (2008). Mitochondrial DNA damage and its potential role in retinal degeneration. *Progress in Retinal and Eye Research*.

[11] Caldwell RB, Bartoli M, Behzadian MA (2005). Vascular endothelial growth factor and diabetic retinopathy: role of oxidative stress. *Current Drug Targets*.

[12] Kowluru RA, Chan P-S (2007). Oxidative stress and diabetic retinopathy. *Experimental Diabesity Research*.

[13] Kaur C, Foulds WS, Ling EA (2008). Blood-retinal barrier in hypoxic ischaemic conditions: basic concepts, clinical features and management. *Progress in Retinal and Eye Research*.

[14] Wigglesworth JM, Baum H, Youdim MBH (1988). Iron dependent enzymes in the brain. *Brain Iron: Neurochemical and Behavioural, Aspects*.

[15] Forge JK, Pedchenko TV, LeVine SM (1998). Iron deposits in the central nervous system of SJL mice with experimental allergic encephalomyelitis. *Life Sciences*.

[16] He X, Hahn P, Iacovelli J (2007). Iron homeostasis and toxicity in retinal degeneration. *Progress in Retinal and Eye Research*.

[17] Halliwell B, Gutteridge JMC (1990). Role of free radicals and catalytic metal ions in human disease: an overview. *Methods in Enzymology*.

[18] Fridovich I (1978). The biology of oxygen radicals. *Science*.

[19] Hahn P, Ying G-S, Beard J, Dunaief JL (2006). Iron levels in human retina: sex difference and increase with age. *NeuroReport*.

[20] Wolozin B, Golts N (2002). Book review: iron and Parkinson’s disease. *The Neuroscientist*.

[21] Honda K, Casadesus G, Petersen RB, Perry G, Smith MA (2004). Oxidative stress and redox-active iron in Alzheimer's disease. *Annals of the New York Academy of Sciences*.

[22] Wong RW, Richa DC, Hahn P, Green WR, Dunaief JL (2007). Iron toxicity as a potential factor in AMD. *Retina*.

[23] Farkas RH, Chowers I, Hackam AS (2004). Increased expression of iron-regulating genes in monkey and human glaucoma. *Investigative Ophthalmology and Visual Science*.

[24] Dawczynski J, Blum M, Winnefeld K, Strobel J (2002). Increased content of zinc and iron in human cataractous lenses. *Biological Trace Element Research*.

[25] Abboud S, Haile DJ (2000). A novel mammalian iron-regulated protein involved in intracellular iron metabolism. *Journal of Biological Chemistry*.

[26] Donovan A, Brownlie A, Zhou Y (2000). Positional cloning of zebrafish ferroportin1 identifies a conserved vertebrate iron exporter. *Nature*.

[27] Patel BN, David S (1997). A novel glycosylphosphatidylinositol-anchored form of ceruloplasmin is expressed by mammalian astrocytes. *Journal of Biological Chemistry*.

[28] Vulpe CD, Kuo Y-M, Murphy TL (1999). Hephaestin, a ceruloplasmin homologue implicated in intestinal iron transport, is defective in the sla mouse. *Nature Genetics*.

[29] Sipe DM, Murphy RF (1991). Binding to cellular receptors results in increased iron release from transferrin at mildly acidic pH. *Journal of Biological Chemistry*.

[30] Hunt RC, Davis AA (1992). Release of iron by human retinal pigment epithelial cells. *Journal of Cellular Physiology*.

[31] Aisen P, Enns C, Wessling-Resnick M (2001). Chemistry and biology of eukaryotic iron metabolism. *International Journal of Biochemistry and Cell Biology*.

[32] Levi S, Santambrogio P, Cozzi A (1994). The role of the L-chain in ferritin iron incorporation. Studies of homo and heteropolymers. *Journal of Molecular Biology*.

[33] Yefimova MG, Jeanny J-C, Guillonneau X (2000). Iron, ferritin, transferrin, and transferrin receptor in the adult rat retina. *Investigative Ophthalmology and Visual Science*.

[34] Hahn P, Qian Y, Dentchev T (2004). Disruption of ceruloplasmin and hephaestin in mice causes retinal iron overload and retinal degeneration with features of age-related macular degeneration. *Proceedings of the National Academy of Sciences of the United States of America*.

[35] Hentze MW, Kühn LC (1996). Molecular control of vertebrate iron metabolism: mRNA-based regulatory circuits operated by iron, nitric oxide, and oxidative stress. *Proceedings of the National Academy of Sciences of the United States of America*.

[36] Beinert H, Kiley P (1996). Redox control of gene expression involving ironsulfur proteins. Change of oxidation-state or assembly/disassembly of Fe-S clusters?. *FEBS Letters*.

[37] Guo B, Phillips JD, Yu Y, Leibold EA (1995). Iron regulates the intracellular degradation of iron regulatory protein 2 by the proteasome. *The Journal of Biological Chemistry*.

[38] Iwai K, Drake SK, Wehr NB (1998). Iron-dependent oxidation, ubiquitination, and degradation of iron regulatory protein 2: implications for degradation of oxidized proteins. *Proceedings of the National Academy of Sciences of the United States of America*.

[39] Rouault TA, Klausner RD (1996). Iron-sulfur clusters as biosensors of oxidants and iron. *Trends in Biochemical Sciences*.

[40] Laicine EM, Haddad A (1994). Transferrin one of the major vitreous proteins, is produced within the eye. *Experimental Eye Research*.

[41] Dernouchamps JP, Vaerman JP, Michiels J, Heremans JF (1975). Transferrin in the intraocular fluids in rabbit. *Ophthalmologica*.

[42] Chowers I, Wong R, Dentchev T (2006). The iron carrier transferrin is upregulated in retinas from patients with age-related macular degeneration. *Investigative Ophthalmology and Visual Science*.

[43] Chowers I, Gunatilaka TL, Farkas RH (2003). Identification of novel genes preferentially expressed in the retina using a custom human retina cDNA microarray. *Investigative Ophthalmology and Visual Science*.

[44] Picard E, Fontaine I, Jonet L (2008). The protective effect of transfferin in Muller glial cells after iron-induced toxicity. *Molecular Vision*.

[45] Bruinink A, Sidler C, Birchler F (1996). Neurotrophic effects of transferrin on embryonic chick brain and neural retina cell cultures. *International Journal of Developmental Neuroscience*.

[46] Guo J-H, Juan S-H, Aust SD (1998). Suppression of cell growth by heavy chain ferritin. *Biochemical and Biophysical Research Communications*.

[47] Cozzi A, Levi S, Corsi B (2003). Role of iron and ferritin in TNF*α*-induced apoptosis in HeLa cells. *FEBS Letters*.

[48] Corsi B, Cozzi A, Arosio P (2002). Human mitochondrial ferritin expressed in HeLa cells incorporates iron and affects cellular iron metabolism. *Journal of Biological Chemistry*.

[49] Drysdale J, Arosio P, Invernizzi R (2002). Mitochondrial ferritin: a new player in iron metabolism. *Blood Cells, Molecules &amp; Diseases*.

[50] Hahn P, Dentchev T, Qian Y, Rouault T, Harris ZL, Dunaief JL (2004). Immunolocalization and regulation of iron handling proteins ferritin and ferroportin in the retina. *Molecular Vision*.

[51] Drysdale J, Goralska M, Ferrell J (2009). Iron metabolism in the eye: a review. *Experimental Eye Research*.

[52] Friedman PA, Zeidel ML (1999). Victory at C. *Nature Medicine*.

[53] Minamizono A, Tomi M, Hosoya K-I (2006). Inhibition of dehydroascorbic acid transport across the rat blood-retinal and -brain barriers in experimental diabetes. *Biological and Pharmaceutical Bulletin*.

[54] Woodford BJ, Tso MOM, Lam KW (1988). Reduced and oxidized ascorbates in guinea pig retina under normal and light-exposed conditions. *Investigative Ophthalmology and Visual Science*.

[55] Barba I, Garcia-Ramirez M, Hernández C Metabolic fingerprints of proliferative diabetic retinopathy . An 1H NMR-based metabonomic approach using vitreous humor.

[56] McGahan MC, Harned J, Grimes AM, Fleisher LN (1994). Regulation of ferritin levels in cultured lens epithelial cells. *Experimental Eye Research*.

[57] Harned J, Grimes AM, McGahan MC (2003). The effect of UVB irradiation on ferritin subunit synthesis, ferritin assembly and Fe metabolism in cultured canine Lens epithelial cells. *Photochemistry and Photobiology*.

[58] Osaki S (1966). Kinetic studies of ferrous ion oxidation with crystalline human ferroxidase (ceruloplasmin). *The Journal of Biological Chemistry*.

[59] Yau K-W, Baylor DA (1989). Cyclic GMP-activated conductance of retinal photoreceptor cells. *Annual Review of Neuroscience*.

[60] Moyseyev G, Takahashi Y, Chen Y (2006). RPE65 is an iron(II)-dependent isomerohydrolase in the retinoid visual cycle. *The Journal of Biological Chemistry*.

[61] Lall MM, Ferrell J, Nagar S, Fleisher LN, McGahan MC (2008). Iron regulates L-cystine uptake and glutathione levels in lens epithelial and retinal pigment epithelial cells by its effect on cytosolic aconitase. *Investigative Ophthalmology and Visual Science*.

[62] Lieth E, Barber AJ, Xu B (1998). Glial reactivity and impaired glutamate metabolism in short-term experimental diabetic retinopathy. *Diabetes*.

[63] Kowluru RA, Engerman RL, Case GL, Kern TS (2001). Retinal glutamate in diabetes and effect of antioxidants. *Neurochemistry International*.

[64] Pulido JE, Pulido JS, Erie JC (2007). A role for excitatory amino acids in diabetic eye disease. *Experimental Diabesity Research*.

[65] Ambati J, Chalam KV, Chawala DK (1997). Elevated *γ*-aminobutyric acid, glutamate, and vascular endothelial growth factor levels in the vitreous of patients with proliferative diabetic retinopathy. *Archives of Ophthalmology*.

[66] Cosso L, Maineri EP, Traverso N (2001). Induction of heme oxygenase 1 in liver of spontaneously diabetic rats. *Free Radical Research*.

[67] Nishimura RN, Dwyer BE, Lu S-Y (1996). Localization of heme oxygenase in rat retina: effect of light adaptation. *Neuroscience Letters*.

[68] Kuesap J, Li B, Satarug S (2008). Prostaglandin D2 induces heme oxygenase-1 in human retinal pigment epithelial cells. *Biochemical and Biophysical Research Communications*.

[69] Sun M-H, Pang J-HS, Chen S-L (2007). Photoreceptor protection against light damage by AAV-mediated overexpression of heme oxygenase-1. *Investigative Ophthalmology and Visual Science*.

[70] Cukiernik M, Mukherjee S, Downey D, Chakabarti S (2003). Heme oxygenase in the retina in diabetes. *Current Eye Research*.

[71] Dawn B, Bolli R (2005). HO-1 induction by HIF-1: a new mechanism for delayed cardioprotection?. *American Journal of Physiology*.

[72] Bianchi L, Tacchini L, Cairo G (1999). HIF-1-mediated activation of transferrin receptor gene transcription by iron chelation. *Nucleic Acids Research*.

[73] Martin F, Linden T, Katschinski DM (2005). Copper-dependent activation of hypoxia-inducible factor (HIF)-1: implications for ceruloplasmin regulation. *Blood*.

[74] Obolensky A, Berenshtein E, Konijn AM, Banin E, Chevion M (2008). Ischemic preconditioning of the rat retina: protective role of ferritin. *Free Radical Biology and Medicine*.

[75] Sulochana KN, Coral K, Punitham R, Sharma T, Kasinatham N, Ramakrishan S (2004). Trace elements iron, copper and zinc in vitreous of patients with various vitroretinal disease. *Indian Journal of Ophthalmology*.

[76] Weller M, Clausen R, Heimann K, Wiedemann P (1990). Iron-binding proteins in the human vitreous: iactoferrin and transferrin in health and in proliferative intraocular disorders. *Ophthalmic Research*.

[77] Weller M, Wiedemann P, Moter H, HeimannK K (1988). Transferrin and transefferin receptor expression in intraocular proliferative disease. APAAP-immunolabeling of retinal membranes and ELISA for vitreal transfferin. *Graefes`s Achieve of Clinical and Experimental Ophthalmology*.

[78] Cussimanio BL, Booth AA, Todd P, Hudson BG, Khalifah RG (2003). Unusual susceptibility of heme proteins to damage by glucose during non-enzymatic glycation. *Biophysical Chemistry*.

[79] Belcher JD, Beckman JD, Balla G, Balla J, Vercellotti G (2010). Heme degradation and vascular injury. *Antioxidants & Redox Signaling*.

[80] Ishizaka N, Saito K, Furuta K (2007). Angiotensin II-induced regulation of the expression and localization of iron metabolism-related genes in the rat kidney. *Hypertension Research*.

[81] Qiuju L, Liang S, Yi T, Guanjun W, Xu L, Lu C (2009). Role of iron deficiency and overload in the pathogenesis of diabetes and diabetic complications. *Current Medicinal Chemistry*.

[82] Nagai N, Noda K, Urano T (2005). Selective suppression of pathologic, but not physiologic, retinal neovascularization by blocking the angiotensin II type 1 receptor. *Investigative Ophthalmology and Visual Science*.

[83] Sarlos S, Rizkalla B, Moravski CJ, Cao Z, Cooper ME, Wilkinson-Berka JL (2003). Retinal angiogenesis is mediated by an interaction between the angiotensin type 2 receptor, VEGF, and angiopoietin. *American Journal of Pathology*.

[84] Wheeler-Schilling TH, Kohler K, Sautter M, Guenther E (1999). Angiotensin II receptor subtype gene expression and cellular localization in the retina and non-neuronal ocular tissues of the rat. *European Journal of Neuroscience*.

[85] Wilkinson-Berka JL (2006). Angiotensin and diabetic retinopathy. *International Journal of Biochemistry and Cell Biology*.

[86] Simó R, Hernández C (2009). Advances in the medical treatment of diabetic retinopathy. *Diabetes Care*.

[87] Danser AHJ, van den Dorpel MA, Deinum J (1989). Renin, prorenin, and immunoreactive renin in vitreous fluid from eyes with and without diabetic retinopathy. *Journal of Clinical Endocrinology and Metabolism*.

[88] Funatsu H, Yamashita H (2003). Pathogenesis of diabetic retinopathy and the renin-angiotensin system. *Ophthalmic and Physiological Optics*.

[89] Gao B-B, Chen X, Timothy N, Aiello LP, Feener EP (2008). Characterization of the vitreous proteome in diabetes without diabetic retinopathy and diabetes with proliferative diabetic retinopathy. *Journal of Proteome Research*.

[90] Rogers BS, Symons RCA, Komeima K (2007). Differential sensitivity of cones to iron-mediated oxidative damage. *Investigative Ophthalmology and Visual Science*.

[91] Charkoudian LK, Dentchev T, Lukinova N, Wolkow N, Dunaief JL, Franz KJ (2008). Iron prochelator BSIH protects retinal pigment epithelial cells against cell death induced by hydrogen peroxide. *Journal of Inorganic Biochemistry*.

[92] Lukinova N, Iacovelli J, Dentchev T (2009). Iron chelation protects the retinal pigment epithelial cell line ARPE-19 against cell death triggered by diverse stimuli. *Investigative Ophthalmology &amp; Visual Science*.

[93] Spaide RF, Ho-Spaide WC, Browne RW, Armstrong D (1999). Characterization of peroxidized lipids in Bruch’s membrane. *Retina*.

[94] Pinazo-Durán MD, Verdejo C, Azorín I, Renau-Piqueras J, Iborra FJ (2000). Colocalization of aldehyde dehydrogenases and Fe/NADPH-induced lipid peroxidation in tissue sections of rat retina. *Ophthalmic Research*.

[95] Yamagishi S-I, Ueda S, Matsui T, Nakamura K, Okuda S (2008). Role of advanced glycation end products (AGEs) and oxidative stress in diabetic retinopathy. *Current Pharmaceutical Design*.

[96] Yefimova MG, Jeanny J-C, Keller N (2002). Impaired retinal iron homeostasis associated with defective phagocytosis in Royal College of surgeons rats. *Investigative Ophthalmology and Visual Science*.

[97] Chen H, Lukas TJ, Du N, Suyeoka G, Neufeld AH (2009). Dysfunction of the retinal pigment epithelium with age: increased iron decreases phagocytosis and lysosomal activity. *Investigative Ophthalmology &amp; Visual Science*.

[98] Liu B-F, Miyata S, Kojima H (1999). Low phagocytic activity of resident peritoneal macrophages in diabetic mice: relevance to the formation of advanced glycation end products. *Diabetes*.

[99] Hammes HP, Bartmann A, Engel L, Wülfroth P (1997). Antioxidant treatment of experimental diabetic retinopathy in rats with nicanartine. *Diabetologia*.

[100] Chen K, Gunter K, Maines MD (2000). Neurons overexpressing heme oxygenase-1 resist oxidative stress-mediated cell death. *Journal of Neurochemistry*.

[101] da Silva J-L, Stoltz RA, Dunn MW, Abraham NG, Shibahara S (1997). Diminished heme oxygenase-1 mRNA expression in RPE cells from diabetic donors as quantitated by competitive RT/PCR. *Current Eye Research*.

[102] Foresti R, Motterlini R (1999). The heme oxygenase pathway and its interaction with nitric oxide in the control of cellular homeostasis. *Free Radical Research*.

[103] Chen S, Mukherjee S, Chakraborty C, Chakrabarti S (2003). High glucose-induced, endothelin-dependent fibronectin synthesis is mediated via NF-*κ*B and AP-1. *American Journal of Physiology*.

[104] Dulak J, Józkowicz A, Foresti R (2002). Heme oxygenase activity modulates vascular endothelial growth factor synthesis in vascular smooth muscle cells. *Antioxidants and Redox Signaling*.

[105] Kartikasari AER, Georgiou NA, Visseren FLJ, van Kats-Renaud H, van Asbeck BS, Marx JJM (2004). Intracellular labile iron modulates adhesion of human monocytes to human endothelial cells. *Arteriosclerosis, Thrombosis, and Vascular Biology*.

[106] Koo S-W, Casper KA, Otto KB, Gira AK, Swerlick RA (2003). Iron chelators inhibit VCAM-1 expression in human dermal microvascular endothelial cells. *Journal of Investigative Dermatology*.

[107] Zhang W-J, Frei B (2003). Intracellular metal ion chelators inhibit TNF*α*-induced SP-1 activation and adhesion molecule expression in human aortic endothelial cells. *Free Radical Biology and Medicine*.

[108] Gnana-Prakasam JP, Thangaraju M, Liu K (2009). Absence of iron-regulatory protein Hfe results in hyperproliferation of retinal pigment epithelium: role of cystine/glutamate exchanger. *Biochemical Journal*.

